# Intratumoral Human Papillomavirus in Advanced Prostate Cancer: Systematic Review, Meta-Analysis, and Real-World Evidence from the Largest Cohort to Date

**DOI:** 10.3390/ijms27177831

**Published:** 2026-09-01

**Authors:** Daniele Brenna, Marialuisa Puglisi, Samantha Epistolio, Jessica Barizzi, Martino Pedrani, Giuseppe Salfi, Giovanna Pecoraro, Fabio Turco, Luigi Tortola, Salvatore Cozzi, Thomas Zilli, Gianmarco Leone, Ursula Vogl, Silke Gillessen, Milo Frattini, Ricardo Pereira Mestre

**Affiliations:** 1 Radiation Oncology, Oncology Institute of Southern Switzerland, Ente Ospedaliero Cantonale, CH 6500 Bellinzona, Switzerland; daniele.brenna@eoc.ch (D.B.); salvatore.cozzi@eoc.ch (S.C.); thomas.zilli@eoc.ch (T.Z.); 2 Medical Oncology, Oncology Institute of Southern Switzerland (IOSI), Ente Ospedaliero Cantonale (EOC), CH 6500 Bellinzona, Switzerland; martino.pedrani@eoc.ch (M.P.); giuseppe.salfi@eoc.ch (G.S.); giovanna.pecoraro@eoc.ch (G.P.); fabio.turco@eoc.ch (F.T.); luigi.tortola@eoc.ch (L.T.); gianmarco.leone@eoc.ch (G.L.); ursula.vogl@eoc.ch (U.V.); silke.gillessensommer@eoc.ch (S.G.); ricardo.pereiramestre@eoc.ch (R.P.M.); 3 Laboratory of Genetics and Molecular Pathology, Institute of Pathology, Ente Ospedaliero Cantonale (EOC), CH 6600 Locarno, Switzerland; samantha.epistolio@eoc.ch (S.E.); jessica.barizzi@eoc.ch (J.B.); milo.frattini@eoc.ch (M.F.); 4 Faculty of Biomedical Sciences, Università della Svizzera Italiana, CH 6900 Lugano, Switzerland; 5 Faculty of Medicine, Geneva University, CH 1211 Geneva, Switzerland

**Keywords:** prostate carcinoma, HPV DNA detection, systematic review, meta-analysis

## Abstract

The etiopathogenesis of prostate cancer (PCa) is complex and involves multiple hormonal and environmental factors. Among these, the role of infectious agents remains controversial. Human papillomavirus (HPV) is known to promote carcinogenesis in epithelial tumors and has also been detected, albeit inconsistently, in PCa tissues. This systematic review, meta-analysis, and real-world cohort study aimed to clarify the prevalence and the prognostic role of intratumoral (IT)-HPV detection in locally advanced and metastatic PCa. We performed a systematic literature search including studies reporting IT-HPV detection in locally advanced (LA-PCa) and metastatic PCa (mPCa). Pooled prevalence estimates were calculated using random-effects models. In parallel, HPV DNA was analyzed using archival tissue in a single-center real-world retrospective cohort of 196 metastatic PCa patients. Twenty-three records met the inclusion criteria, comprising 22 full-text publications and one abstract. Nineteen datasets, encompassing 837 patients, contributed to the quantitative synthesis of stage III–IV disease. Pooled IT-HPV prevalence in mPCa was 18.5% (95% CI, 6.4–34%), while including also LA-PCa was 14.6% (95% CI, 5.2–26.8). Eastern countries showed higher IT-HPV prevalence in both mPCa and LA-PCa (40% and 34.3%, respectively) compared with Western regions (0.8% and 0.3%; *p* < 0.0001). HPV-positive PCa was associated with a higher Gleason score (>7) in advanced stage (pooled OR 6.32, 95% CI, 3.13–12.78) compared with IT-HPV-negative PCa; however, no studies reported data on its association with survival outcomes. Within our retrospective cohort, IT-HPV DNA was detected in 3.1% of cases (6/196) and exhibited typical PCa driver alterations, such as TP53, SPOP, PIK3CA, and AR amplification. IT–HPV seems to be infrequent in metastatic and locally advanced PCa in Western populations and more prevalent in Asian cohorts and might be associated with a higher Gleason score. IT–HPV-positive advanced PCa seems to exhibit typical PCa driver alterations. Data on the association between IT–HPV status and survival outcomes in PCa are lacking. Standardized molecular assays and multicenter prospective studies are needed to delineate its significance in PCa.

## 1. Introduction

Prostate cancer (PCa) is the second most frequently diagnosed malignancy among men worldwide, with over 1.4 million new cases diagnosed every year [1,2]. Patients with mPCa have an unfavorable prognosis, with five-year survival rates of 36–43% [3].

The etiopathogenesis of PCa is complex and not yet fully understood, involving genetic susceptibility, hormonal and metabolic factors, and environmental exposures [4].

Recent studies have increasingly focused on the role of chronic inflammation and infectious agents in prostate carcinogenesis. Chronic prostatitis and sexually transmitted infections have been identified as cofactors that induce inflammatory responses, cellular damage, and oxidative stress, potentially facilitating malignant transformation [5]. Within this context, viral infections, particularly those caused by oncogenic viruses, have been hypothesized to contribute to prostate tumorigenesis [6].

Viral infections contribute to roughly 12% of the global cancer burden, with human papillomavirus (HPV) and Epstein–Barr virus (EBV) representing the predominant etiologic agents, accounting for 38% of virus-associated cases [7]. HPV is one of the most common sexually transmitted pathogens and a well-established cause of several epithelial cancers, including cervical, anal, penile, and head and neck carcinomas [8].

HPV frequently causes chronic inflammation of the genitourinary epithelium. While its oncogenic role in cervical and penile cancers is well recognized, emerging evidence from studies examining the genitourinary microbiota suggests a broader involvement in bladder and prostatic tumorigenesis [9,10]. Although PCa is not considered a classical HPV–related neoplasm, the detection of HPV DNA and oncoproteins has prompted further investigations into the potential role of viral infection in its etiopathogenesis [11]. HPV may promote prostate carcinogenesis through E6/E7-mediated inactivation of TP53 and RB1, activation of NF-κB and pro-inflammatory cytokines, leading to chronic inflammation, angiogenesis, and apoptosis resistance [10,11,12,13].

However, current evidence remains controversial, with studies reporting highly variable prevalence rates of HPV in prostate specimens, likely due to methodological and geographic heterogeneity. Clarifying this potential association is of both scientific and clinical interest, as it may reveal novel pathogenic pathways and preventive opportunities. Moreover, although the role of HPV has been extensively investigated in localized PCa [10,11,12,13], its prevalence and prognostic impact in metastatic disease and in LA-PCa remain poorly defined. Available data are limited to small, single-center cohorts, and no systematic synthesis has been published to date.

In this study, we address this gap by combining a comprehensive systematic review and meta-analysis with original clinical data from our retrospective cohort of patients with metastatic PCa, in order to clarify both the prevalence and prognostic relevance of IT–HPV in advanced disease.

## 2. Methods

### 2.1. Systematic Review and Meta-Analysis

#### 2.1.1. Evidence Acquisition

This systematic review and meta-analysis was conducted and reported in accordance with the Preferred Reporting Items for Systematic Reviews and Meta-Analyses (PRISMA) 2020 guidelines. The study protocol was registered in PROSPERO (registration number: 1115050).

#### 2.1.2. Search Strategy

We undertook a systematic search of the literature up to 11 May 2026. The search was conducted across one bibliographic database (PubMed) and two citation databases (Scopus and Web of Science). The search strings and the search strategy can be found in Section S1 of the Supplementary Materials.

#### 2.1.3. Study Selection

We conducted a systematic review encompassing observational studies. Eligible designs were cohort, case–control, and cross-sectional studies that evaluated the presence of IT-HPV in tissue from either the primary tumor or metastatic sites of patients with advanced PCa. Studies were required to include patients with metastatic, locally advanced disease or both. Studies could evaluate HPV presence on both formalin-fixed paraffin-embedded (FFPE) blocks and fresh/fresh-frozen tissue. Specimens could be obtained through both surgical resections and biopsies. The selected studies were required to report the number of patients with metastatic or locally advanced disease or provide sufficient data to calculate HPV prevalence in these populations. Two investigators (D.B. and ML.P.) independently performed initial screening of all published manuscripts. Inclusion and exclusion criteria are provided in Section S2 of the Supplementary Materials.

#### 2.1.4. Data Extraction

Two authors (D.B. and ML.P.) independently extracted data, with any disagreements resolved through discussion with a third author (G.S.) to reach a consensus. Collected study characteristics included the author, year, design, geographic area, number of metastatic or locally advanced disease patients in the study population, HPV detection method employed, HPV genotypes detected and their oncogenic-risk category (high-risk or low-risk, where available), IT prevalence of HPV, site of the specimen, Gleason score (GS), other molecular alterations, progression-free survival and overall survival of the HPV-positive cohort. For both advanced and metastatic disease cohorts, data were extracted for each subgroup separately. These subgroups were subsequently treated as independent datasets in all analyses, including the risk of bias assessment.

#### 2.1.5. Statistical Analysis

We conducted a meta-analysis of proportions to estimate the pooled prevalence of IT-HPV DNA in patients with advanced and mPCa, following established methods for single-arm prevalence synthesis as outlined by Barendregt et al. [14]. Study-level proportions (HPV-positive samples/total evaluable samples) were extracted directly from each report. To stabilize variance and appropriately handle extreme proportions, the primary analysis used the Freeman–Tukey double arcsine transformation, with pooled estimates back-transformed and presented with 95% confidence intervals (CIs) and 95% prediction intervals (PIs).

The primary analyses were prespecified as stage-specific: (i) metastatic disease (Stage IV only) and (ii) locally advanced and metastatic combined (Stages III–IV). Study estimates and pooled effects were summarized with forest plots. Between-study heterogeneity was assessed using the Cochran Q statistic and quantified with the I^2^ statistic [15,16]. Given the limited study pool and anticipated clinical and methodological heterogeneity, we applied a random-effects model with restricted maximum likelihood (REML) [17] to derive pooled prevalence and PIs.

We also evaluated the association between IT–HPV and higher GS, defined as >7. When available, we extracted the distribution of GS categories (>7 vs. ≤7) by HPV status and computed study-level ORs for Gleason score > 7 in HPV-positive versus HPV-negative cases. These ORs were then pooled using random-effects REML models, with 95% CIs and heterogeneity metrics reported in parallel with the OR analyses.

Small-study effects and potential publication bias were explored via funnel plot asymmetry; Egger’s regression test was performed when ≥10 studies were available [18]. All analyses were conducted in R (v4.5.1; R Foundation for Statistical Computing, Vienna, Austria) using standard meta-analysis packages for proportions.

In addition to external studies, our single-center cohort from the Oncology Institute of Southern Switzerland (IOSI) met inclusion criteria and was included in the meta-analysis as a discrete dataset.

#### 2.1.6. Risk of Bias Assessment

Two authors (D.B. and G.S.) independently evaluated the quality of included studies using the Newcastle-Ottawa Scale for case–control studies. For case-series studies, we applied the dedicated Joanna Briggs Institute (JBI) critical appraisal tool. The risk of bias graphics were created separately for each assessment tool using the package “robvis” in R v4.4.2 (R Foundation for Statistical Computing, Vienna, Austria) [19].

### 2.2. Retrospective Cohort

A single-center retrospective study was conducted using data extracted from electronic medical records of consecutive patients with mPCa treated at IOSI between 1 January 2014 and 31 December 2023. One hundred ninety-six patients were identified through institutional tumor boards and pathology archives. All cases with FFPE (formalin-fixed paraffin-embedded) tumor tissue available (primary prostate biopsy or prostatectomy; or, when available, metastatic biopsy) and adequate DNA quantity/quality for HPV testing were eligible. Patients without sufficient tumor material, with inadequate DNA quality control, indeterminate histology, or pure small-cell/neuroendocrine carcinoma at presentation were excluded. The present institutional cohort was also included as a discrete dataset in the quantitative synthesis (meta-analysis) of proportions.

#### 2.2.1. Study End Points

Primary objectives were to (i) determine the prevalence of intratumoral HPV DNA detection in patients with locally advanced and metastatic PCa; and (ii) evaluate geographical differences in HPV prevalence in locally advanced PCa patients and in locally advanced and metastatic PCa patients between Eastern (Asia) and Western (Europe, North and South America, Australia) countries, including our Swiss cohort.

The secondary objective was to investigate potential correlations between HPV DNA detection status and PCa prognosis:

- Progression-free survival (PFS) and overall survival (OS) outcomes of IT–HPV-positive versus HPV-negative cohorts;
- Association between IT-HPV status and Gleason score (GS).

The methodology for the statistical analyses performed within the retrospective cohort is detailed in Section S3 of the Supplementary Materials.

#### 2.2.2. HPV Detection Assay

An experienced pathologist of the Pathology Clinic of the Institute of Integrated Diagnostics of Italian Switzerland (IDISI) evaluated the quality and tumor cell content of the FFPE samples. Genomic DNA was extracted from three 8-μm-thick serial sections of each selected FFPE tissue block using the QIAamp DNA FFPE Tissue Kit (QIAGEN LLC, Germantown, MD, USA), according to the manufacturer’s instructions. Extracted DNA was analyzed for the detection of HPV DNA and for HPV genotyping using the Allplex™ HPV28 Detection assay (Seegene Inc., Seoul, Republic of Korea). This assay detects HPV DNA and therefore identifies the presence of viral genetic material but does not, by itself, demonstrate transcriptionally active HPV infection or HPV–driven carcinogenesis. The assay is validated for cytological samples and has also been used for the evaluation of histological specimens [20]. All procedures were performed according to the manufacturer’s instructions.

#### 2.2.3. Statistical Analysis

The primary endpoint—the prevalence of HPV DNA detection—was estimated as the proportion of HPV DNA-positive tumors among evaluable cases, with exact (Clopper–Pearson) 95% confidence intervals. Given the limited number of HPV-positive samples, analyses were restricted to prevalence estimation and descriptive summaries of baseline clinicopathologic and genomic features (including HPV genotype distribution, Next-generation sequencing (NGS) detected alterations, age, prostate-specific antigen (PSA) at diagnosis, GS, disease volume according to CHAARTED criteria, first-line treatment and overall survival). Continuous variables were summarized as medians with interquartile ranges, and categorical variables as counts and percentages.

Survival outcomes were evaluated using the Kaplan–Meier method to estimate survival probabilities. For comparative subgroup analysis, patients were stratified based on HPV status. Patients with non-evaluable HPV status were excluded from stratified comparisons, resulting in a positive cohort (n = 6, comprising all high-risk and low-risk variants) and a negative cohort (n = 181).

Four distinct time-to-event endpoints were analyzed:

1. Overall Survival from Primary Diagnosis (OS–Primary): Time from the date of primary tumor diagnosis to death from any cause.
2. Progression-Free Survival from Primary Diagnosis (PFS–Primary): Time from primary diagnosis to the first event of disease progression (occurring during the metastatic phase) or death.
3. Overall Survival from mHSPC (OS–mHSPC): Time from the diagnosis of metastatic hormone-sensitive prostate cancer (mHSPC) to death from any cause.
4. Progression-Free Survival from mHSPC (PFS-mHSPC): Time from the initiation of first-line mHSPC therapy to disease progression or death.

Patients who did not experience an event were censored at the date of their last follow-up. Patients with missing essential dates were excluded from specific analyses. Differences between groups were assessed using the Log–rank test (*p* < 0.05). Median survival times and interquartile ranges (IQR, 25th–75th percentiles) were calculated.

All analyses were conducted in R v4.5.1 (R Foundation for Statistical Computing, Vienna, Austria).

#### 2.2.4. Ethics and Consent

The study was conducted in accordance with the 1964 Declaration of Helsinki and its later amendments. The protocol fell within an approved retrospective data collection framework by the local ethics committee (2023-01208; CE 4401) on 19 July 2023. All living participants provided general or study-specific informed consent for use of clinical data and leftover tissue for research. For deceased patients, data use followed local regulations and ethics approval conditions. Identifiable information was removed prior to analysis.

## 3. Results

### 3.1. Literature Search and Study Selection

**Figure 1:**
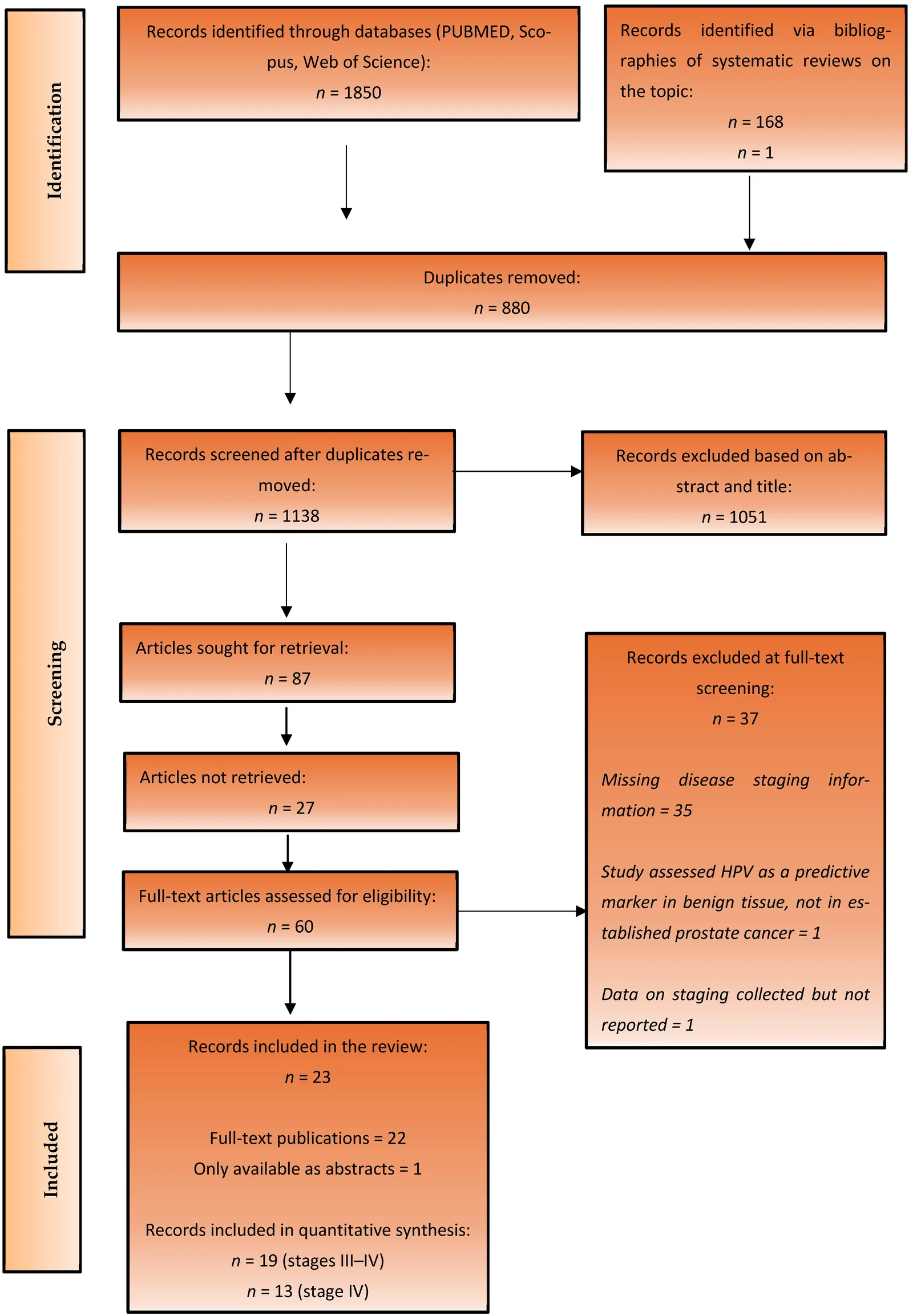
PRISMA 2020 flow diagram of study identification and selection. The diagram summarizes records identified through database searches and reference-list screening, duplicate removal, title/abstract screening, full-text assessment, reasons for exclusion, and studies included in the systematic review and quantitative syntheses.

The systematic literature search across PubMed, Scopus, and Web of Science initially identified 1850 records. An additional 168 records were identified through the manual screening of reference lists from relevant systematic reviews. After the removal of duplicates, a total of 1138 unique records were screened. Ultimately, 23 records were included in the final systematic review, comprising 22 full-text publications and one record available only as an abstract. The study selection process is summarized in the PRISMA flow diagram (Figure 1).

### 3.2. Characteristics of Included Studies

Among the selected studies, a significant geographical heterogeneity was observed, aligning with the stratification used in our subgroup analysis: 10 records (43.5%) originated from Eastern countries [7,10,21,22,23,24,25,26,27,28] and 13 records (56.5%) from Western countries [5,29,30,31,32,33,34,35,36,37,38,39,40].

The majority of studies (n = 14) had a retrospective case–control design. The remaining studies included prospective case–control (n = 2) [35,39], cross-sectional (n = 2) [5,24], and descriptive case series (n = 5) [29,33,34,37,38,40]. The tissue samples analyzed were predominantly derived from the primary tumor, obtained through radical prostatectomy, TURP, or biopsy. Only three studies explicitly analyzed samples from metastatic sites [21,22,29]. FFPE tissue was the most common sample type (82.6% of studies), followed by fresh-frozen tissue (13%). HPV detection methodologies evolved significantly over time, with earlier studies relying on standard PCR and Southern blot [29], while more recent works employed highly sensitive methods like quantitative PCR [31], genotyping arrays [34,39], and NGS [28,40]. The detailed characteristics of each included study are summarized in Supplementary Table S1.

HPV genotype distribution was heterogeneous across the included studies. Among advanced-stage cases for whom genotype-specific data were explicitly reported, high-risk HPV (HR-HPV) types predominated. HPV-16 was detected in 20 cases, HPV-18 in 17, HPV-33 in four, and HPV-58 in one, whereas the low-risk genotype HPV-6 was detected in one case. Additional HPV genotypes, including HPV-31, HPV-42, HPV-11, HPV-54, and HPV-91, were reported in some of the overall study populations; however, their distribution within the advanced-disease subgroup could not be determined from the published data. In our institutional cohort, five of six HPV DNA-positive tumors harbored HR-HPV genotypes (HPV-16, n = 4; HPV-66, n = 1), whereas one harbored the low-risk genotype HPV-6. Because genotype-specific results were inconsistently reported across studies and were frequently unavailable separately for the advanced-stage subgroup, a formal pooled prevalence analysis according to HPV genotype or oncogenic-risk category was not considered methodologically appropriate.

Of the 23 records included in the systematic review, five could not be included in any quantitative synthesis as they did not report poolable prevalence data for the target cohorts [5,7,31,33,35].

The meta-analyses were therefore conducted on 18 published records together with our institutional cohort (19 total). The Advanced Stage (Stage III–IV) analysis included all 19 records; the Stage IV analysis included 13 datasets (12 published studies plus our cohort) [23,25,30,32,37,40]. Data on PFS or OS specifically comparing IT-HPV-positive and IT-HPV-negative populations, together with the corresponding HRs, were not available in any of the included studies; therefore, a meta-analysis of survival outcomes was not performed.

### 3.3. Meta-Analysis of HPV Prevalence in Metastatic Prostate Cancer

**Figure 2:**
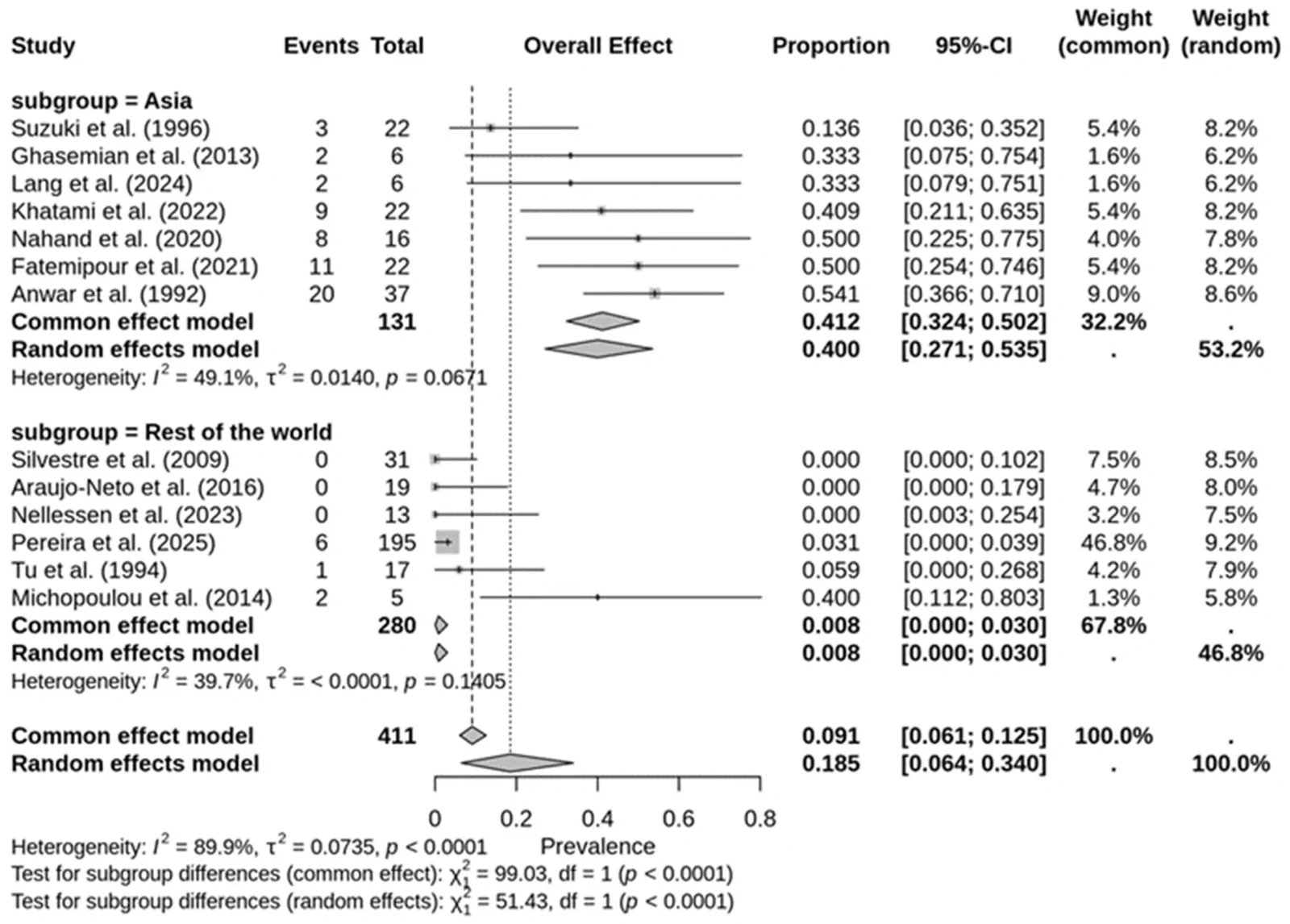
Meta-analysis of intratumoral HPV DNA prevalence in metastatic prostate cancer. Forest plot showing study-specific and pooled prevalence estimates with 95% confidence intervals using a random-effects model, together with subgroup analysis according to geographical region (Eastern versus Western countries). The published studies shown in the forest plot correspond to references [10,21,22,24,26,27,28,29,34,36,38,39]; Pereira et al. (2025) represents the present institutional cohort. Between-study heterogeneity is reported using the I^2^ statistic. HPV, human papillomavirus; CI, confidence interval.

Meta-analysis focused on patients with metastatic disease included 13 studies, encompassing 411 patients. The pooled prevalence of HPV in this cohort was 18.5% (95% CI, 6.4–34.0%) with high heterogeneity across included studies (I^2^ = 89.9%, *p* < 0.01) (Figure 2). A subgroup analysis by geographical region showed that the prevalence was markedly higher in studies from Eastern countries (40%, 95% CI, 27.1–53.5%) compared to those from Western countries (0.8%, 95% CI, 0–3.0%), *p* < 0.0001 (Figure 2).

### 3.4. Meta-Analysis of HPV Prevalence in Locally Advanced and Metastatic Stage (III-IV) PCa

**Figure 3:**
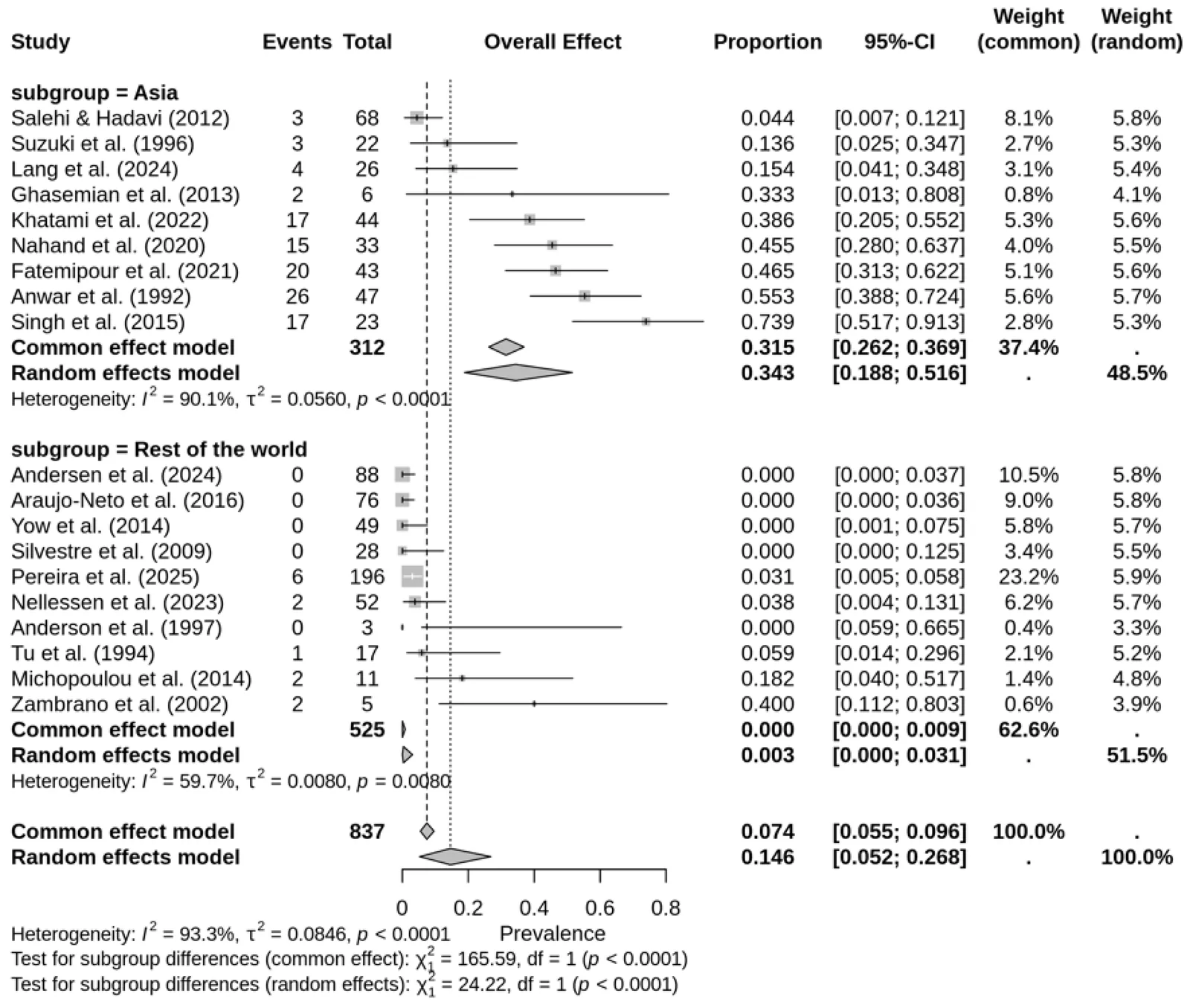
Meta-analysis of intratumoral HPV DNA prevalence in locally advanced and metastatic prostate cancer (stages III–IV). Forest plot showing study-specific and pooled prevalence estimates with 95% confidence intervals using a random-effects model, together with subgroup analysis according to geographical region (Eastern versus Western countries). The published studies shown in the forest plot correspond to references [10,21,22,23,24,25,26,27,28,29,30,32,34,36,37,38,39,40]; Pereira et al. (2025) represents the present institutional cohort. Between-study heterogeneity is reported using the I^2^ statistic. HPV, human papillomavirus; CI, confidence interval.

A second, broader meta-analysis was performed to include patients with both locally advanced and metastatic disease. This analysis included 19 studies encompassing 837 patients. The pooled prevalence of HPV was 14.6% (95% CI, 5.2–26.8%), with high heterogeneity (I^2^ = 93.3%, *p* < 0.01) (Figure 3).

The geographical subgroup analysis showed a prevalence of 34.3% (95% CI, 18.8–51.6%) in Eastern countries versus 0.3% (95% CI, 0–3.1%) in the Western ones. Heterogeneity remained high in the Eastern subgroup (90.1%) but was substantially lower (59.7%) in the Western cohort (*p* < 0.0001) (Figure 3).

### 3.5. Association Between Gleason Score and HPV DNA Detection in Advanced-Stage PCa

**Figure 4:**
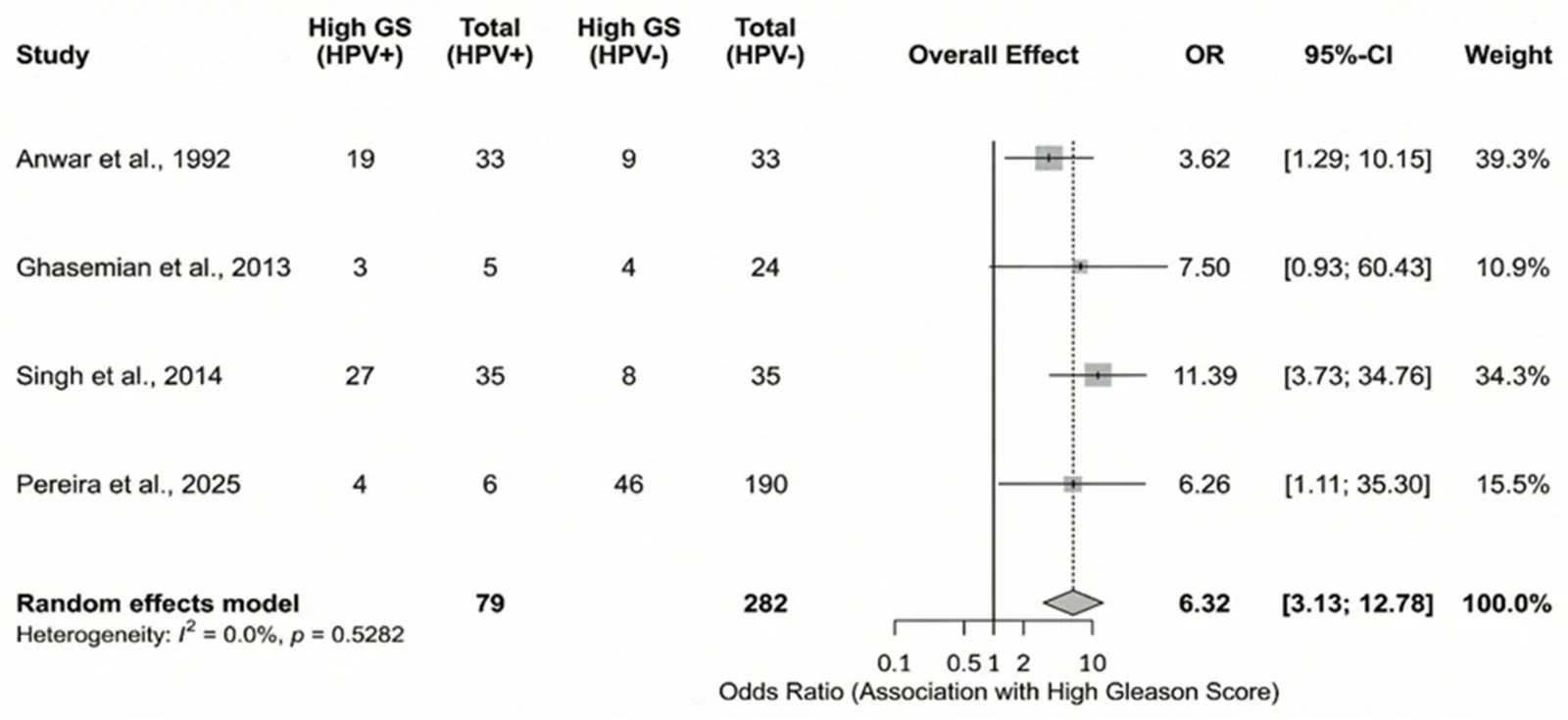
Association between intratumoral HPV DNA detection and high Gleason score in advanced prostate cancer. Forest plot showing study-specific and pooled odds ratios for Gleason score > 7 in HPV DNA-positive versus HPV DNA-negative tumors using a random-effects model. The published studies shown in the forest plot correspond to references [21,24,25]; Pereira et al. (2025) represents the present institutional cohort. Squares represent study-specific estimates and horizontal lines their 95% confidence intervals; the diamond represents the pooled estimate. HPV, human papillomavirus; GS, Gleason score; OR, odds ratio; CI, confidence interval.

We next evaluated whether HPV detection is associated with more aggressive pathological features in advanced PCa. By pooling the available evidence and including our Swiss real-world cohort, we observed a consistent association between HPV detection and higher GS (>7). In a meta-analysis including three eligible studies plus our cohort, HPV DNA-positive advanced PCa was significantly more likely to present with GS > 7 (pooled OR 6.32, 95% CI, 3.13–12.78) (Figure 4).

The potential contribution of oncogenic HPV types was also considered. Among the studies contributing to this analysis, high-risk HPV genotypes were investigated in several cohorts; however, genotype-specific clinicopathological data were not consistently available for all HPV-positive cases. Therefore, a separate meta-analysis restricted exclusively to HR-HPV-positive tumors could not be reliably performed. In our institutional cohort, five of the six HPV DNA-positive tumors harbored HR-HPV genotypes (HPV-16 or HPV-66), whereas one harbored the low-risk genotype HPV-6. Clinicopathological characteristics of the HR-HPV-positive cases are described in the Clinical and Molecular Characteristics of HPV DNA-Positive Patients in the Swiss Cohort Section and Table 1.

### 3.6. Clinicopathological and Genomic Landscape of the Real-World Metastatic Cohort

Our original retrospective cohort included 196 patients with mPCa. The median age at diagnosis was 69.67 years (IQR, 62.72–76). At the time of metastatic diagnosis, the median PSA was 29.35 ng/mL (IQR, 9.97–94.12).

Median OS from mHSPC diagnosis (n = 195) was 86.8 months (IQR, 41.9–114.4) in the overall population, 86.8 months in the HPV-negative group, and 92.1 months in the HPV-positive group, with no statistically significant difference (*p* = 0.642) (Figure S1B in Supplementary Materials).

The median PFS calculated from the initiation of first-line mHSPC therapy (n = 194) was 32.8 months (IQR, 15.7–77.3) (Figure S1D in Supplementary Materials). Stratified analysis yielded a median PFS of 35.2 months for HPV-negative patients and 29.1 months for HPV-positive patients (*p* = 0.816) (Figure S1D in Supplementary Materials).

Additional survival analyses for the metastatic PCa cohort are provided in the Supplementary Materials; Figure S1A,C show Kaplan–Meier estimates of overall survival and progression-free survival from primary diagnosis, respectively, in the overall population and stratified by HPV status, with no statistically significant differences observed between HPV-positive and HPV-negative patients.

Considering the clinical and molecular characteristics of the 196 patients, all variables are comprehensively summarized in Supplementary Table S2. One hundred and twenty patients (61.2%) presented with de novo disease, 75 (38.3%) had metachronous disease, and one patient (0.5%) had locally advanced disease.

According to the CHAARTED criteria, patients were classified as high volume in 109 cases (55.6%) and low volume in 87 (44.4%).

Since this was a historical cohort of patients treated between 2014 and 2023, treatment patterns should be interpreted in the context of the major changes that occurred in international guidelines for metastatic prostate cancer during this period. Overall, 72 patients (36.7%) received androgen deprivation therapy (ADT) alone as first-line systemic therapy for metastatic disease. Ninety-three patients (47.4%) were treated with ADT plus an androgen-receptor pathway inhibitor (ARPI), while 15 (7.7%) received ADT plus chemotherapy and 13 (6.6%) received triplet therapy with ADT, ARPI, and docetaxel.

Tissue samples for NGS were most frequently obtained from prostate (151 cases, 77%), followed by bone (11 cases, 5.6%), lymph nodes (11 cases, 5.6%) and liver (10 cases, 5.1%). Analysis of microsatellite instability (MSI) assessed on 189 patients revealed that 7 patients (3.7%) had high instability (MSI-H), while the vast majority of the cohort, 182 patients (96.3%), were classified as microsatellite stable (MSS).

The NGS analysis identified a wide range of genomic alterations and was performed on primary prostate tumor tissue in 151 patients (77.0%), on metastatic tissue in 41 patients (20.9%), and on blood-based samples in 2 patients (1.0%). In 2 additional cases (1.0%), both primary tumor and metastatic tissue were reported as the source of NGS material. The most frequently altered gene was TP53, altered in 50 patients (28.1%). Alterations in the PTEN/PI3K pathway were the second most common finding, identified in 22 patients (12.4%), followed by alterations in SPOP in 17 patients (9.6%). Other recurrent alterations included those in DDR (21 patients, 11.8%), MYC, AR, and CTNNB1, which were observed with equal frequency, each being identified in 8 patients (4.5% of the cohort).

#### Clinical and Molecular Characteristics of HPV DNA-Positive Patients in the Swiss Cohort

For 187 patients (95.4%), we could obtain definitive results on the presence of HPV DNA in their tissue biopsies. Six patients (3.1%) were HPV-positive and 181 (92.3%) were HPV-negative. In the remaining nine patients (4.6%), the analyses yielded inconclusive results.

The detailed patient-specific clinical and molecular characteristics of all six cases are reported in Table 1. Among the six HPV DNA-positive cases, five harbored high-risk HPV genotypes, specifically HPV-16 and HPV-66, whereas one harbored the low-risk genotype HPV-6. The five HR–HPV-positive patients included two patients with de novo metastatic disease and three with metachronous metastatic disease; three had high-volume and two had low-volume disease. Given the very small number of HR-HPV-positive patients, no inferential comparison of clinicopathological or survival outcomes according to HPV oncogenic-risk category was performed. Considering IT-HPV-positive PCa patients, a diverse set of known PCa driver alterations was identified, excluding two cases with results not available due to insufficient material: one case with a TP53 mutation, two cases with SPOP mutations, and one case with a PIK3CA mutation. Clinical outcomes were equally heterogeneous, with OS from metastatic diagnosis ranging from approximately 8 months to over 90 months.

The single patient with low-risk HPV detection presented with high-volume, metachronous metastatic disease, with an OS of approximately 37.5 months.

### 3.7. Risk of Bias

The risk of bias (RoB) was evaluated for all included studies. The RoB summary tables and traffic light plots can be found in Supplementary Materials (Results S1, Figures S2–S4).

### 3.8. Assessment of Publication Bias

Publication bias was assessed for the two primary meta-analyses using funnel plots and Egger’s linear regression test. Egger’s linear regression test yielded a *p*-value of 0.062 for the ‘Advanced Stage’ analysis and a *p*-value of 0.075 for the ‘Metastatic (Stage IV)’ analysis. Detailed results and funnel plots are available in Supplementary Materials (Results S2, Figures S5 and S6).

## 4. Discussion

To the best of our knowledge, this is the first systematic review and meta-analysis specifically focused on locally advanced and metastatic prostate cancer, integrating published data with a real-world cohort to characterize the prevalence of IT-HPV and its association with adverse pathological features and survival outcomes. The data from this review indicate a complex and heterogeneous association, with significant geographical variation.

A recently published systematic review and meta-analysis by Botting-Provost et al. provides complementary evidence regarding the association between HPV and prostate cancer [41]. Their analysis included 84 studies across different disease settings and HPV ascertainment methods and primarily addressed the epidemiological association between HPV detection and prostate cancer. Among studies assessing HPV DNA in prostate tissue, HPV detection was significantly associated with prostate cancer (pooled OR 2.35, 95% CI, 1.87–2.95), with a stronger association for high-risk HPV types (OR 2.75, 95% CI, 1.69–4.46). However, their study did not specifically focus on locally advanced or metastatic disease and did not perform a dedicated geographical meta-analysis comparing Eastern and Western populations. Our study therefore addresses a complementary question by specifically evaluating intratumoral HPV prevalence in advanced disease, including geographical heterogeneity, pathological aggressiveness, survival outcomes, and molecular characteristics in a real-world metastatic cohort.

Our meta-analysis revealed a pooled HPV prevalence of 18.5% in metastatic PCa and 14.6% in stage III–IV disease, with substantial between-study heterogeneity. A marked geographical difference was observed, with considerably higher HPV DNA detection rates in Eastern than in Western cohorts (40% vs. 0.8% in metastatic disease and 34.3% vs. 0.3% in stage III–IV disease). Similar geographical heterogeneity has previously been reported in prostate cancer meta-analyses [11,12,42,43]. Importantly, the novelty of the present study does not lie in identifying geographical heterogeneity per se, which has already been described, but in demonstrating that this pattern remains evident when the analysis is specifically restricted to locally advanced and metastatic PCa.

These findings are consistent with previous meta-analyses reporting an association between HPV detection and prostate cancer risk [44,45,46].

A comparable geographical signal has also been observed in other genitourinary malignancies. In a systematic review and meta-analysis of bladder cancer, Khatami et al. reported no statistically significant overall association between HPV and bladder cancer (OR 2.08, 95% CI, 0.94–4.59), but subgroup analysis showed a markedly stronger association in Asian populations (OR 6.29, 95% CI, 2.17–18.25) [47]. The authors suggested that genetic and ethnic background, lifestyle, sexual behavior, and other unidentified factors may contribute to this geographical heterogeneity. These findings support the concept that regional differences in HPV-associated genitourinary malignancies are likely multifactorial rather than attributable to a single biological mechanism.

Several additional factors may contribute to the East–West differences observed in our analysis, including variation in background HPV epidemiology and genotype distribution, host susceptibility, study populations, tissue sampling and preservation, PCR primers, molecular detection platforms, analytical sensitivity, and the potential for contamination in highly sensitive assays [48,49]. Therefore, the observed geographical difference should not be interpreted as evidence of a distinct HPV-driven form of PCa in Eastern populations. The relatively small number of HPV DNA-positive cases identified in Western cohorts further limits the precision of this comparison and supports a cautious, hypothesis-generating interpretation.

Differences in HPV vaccination coverage between Eastern and Western countries may also influence contemporary HPV epidemiology. However, vaccination is unlikely to fully explain the geographical pattern observed in the present analysis. Patients with locally advanced or metastatic PCa generally belong to older birth cohorts whose potential HPV exposure would have occurred before the widespread implementation of HPV vaccination, particularly male vaccination. Nevertheless, increasing vaccination coverage may modify HPV prevalence and potentially HPV-associated disease patterns in future generations.

Our single-centre Swiss cohort comprised 196 patients and represents the largest dataset to date reporting IT-HPV detection in metastatic PCa. We observed a low HPV DNA prevalence (3.1%), with only six positive cases, a finding consistent with the low detection rates reported in other Western cohorts. The detection of HPV-16, the most common genotype in our cohort, is particularly relevant, as HPV-16 has been identified as the most significant high-risk genotype associated with PCa risk [43]. Furthermore, the detection of additional high-risk (HPV-66) and low-risk (HPV-6) types in our cohort is consistent with previous systematic reviews, such as Moghoofei et al., reporting a broad spectrum of HPV genotypes in prostate tissues [45].

To our knowledge, this is the largest Western study investigating HPV detection in mPCa using a highly sensitive real-time PCR–based assay for viral detection in tumor tissue. IT-HPV-positive tumors harbored canonical PCa driver alterations, including TP53 and SPOP mutations, and PIK3CA activation. This finding is notably in contrast with some key molecular studies included in our systematic review. For example, Lang et al. reported that HPV-positive tumors in their cohort possessed a unique genomic landscape with fewer mutations in common driver genes like TP53 and SPOP, while Suzuki et al. observed a mutually exclusive trend between HPV-16 positivity and TP53 mutations [22,28].

Our systematic review and meta-analysis support an association between IT–HPV detection and a more aggressive pathological phenotype [21,25]. By integrating the published evidence with our real-world cohort in a dedicated meta-analysis of GS distribution, we provide consolidated evidence that IT-HPV-positive advanced PCa is more likely to present with high-grade disease. Moreover, the only study focused on investigating survival, Pascale et al. (2013), reported a significantly worse OS in HPV-positive patients [50].

These aggressive clinical features are possibly supported by a molecular rationale. Beyond the classic inactivation of TP53 and RB1 by the E6 and E7 oncoproteins, recent studies have highlighted pathways that directly promote aggressiveness and metastasis [51]. Fatemipour et al. demonstrated that HPV oncoproteins can induce the epithelial–mesenchymal transition (EMT), a key process for metastatic dissemination [26]. Furthermore, Nahand et al. reported that HPV-related mechanisms may promote resistance to anoikis, or detachment-induced cell death, thereby promoting cell survival during the metastatic cascade [7]. Other proposed mechanisms include the creation of a pro-tumorigenic microenvironment via chronic inflammation (IL-6, STAT3) [10] and the oncogenic reprogramming of the cell through the alteration of microRNA (miRNA) profiles [27].

Importantly, HPV DNA detection alone does not establish transcriptionally active infection or an etiological role in prostate carcinogenesis. The physical state and biological activity of HPV in prostate tissue have been investigated in a limited number of studies. Evidence of viral biological activity has been reported, including HPV E7 oncoprotein expression and HPV-related gene expression in prostate cancer tissues. Pascale et al. reported nuclear E7 oncoprotein expression in HPV–positive prostate cancers [50]. However, these biological assessments have been performed in only a limited number of studies, and the available evidence remains insufficient to establish a causal role of HPV in prostate carcinogenesis.

The physical status of HPV DNA may also be biologically relevant. Viral genomes can be present in episomal or integrated forms, and integration may promote deregulation of viral oncogene expression. Nevertheless, available evidence regarding HPV integration in prostate cancer remains limited and heterogeneous, and neither HPV DNA detection nor integration alone is sufficient to establish a causal role in prostate carcinogenesis. Future studies should therefore combine HPV DNA genotyping with assessment of viral physical status, E6/E7 transcription and/or protein expression, and spatial localization of viral signals within malignant cells. Such approaches will be necessary to distinguish incidental HPV DNA detection from biologically active and potentially oncogenic HPV involvement.

While this study provides new insights, several limitations must be acknowledged. The low number of HPV-positive cases restricts the generalizability of our findings, and the predominance of retrospective designs among included studies introduces potential biases. Furthermore, most available data derive from primary tumor samples, with limited evaluation of metastatic tissues, an area that warrants further exploration given the high heterogeneity of PCa biology across different disease locations [52]. Future research should focus on large, prospective multicenter cohorts incorporating advanced molecular profiling, integration of microbiome analyses, and standardized detection methods to clarify the potential role of HPV and the genitourinary microbiota in prostate carcinogenesis.

## 5. Conclusions

In conclusion, our systematic review reveals a marked geographical disparity, with Eastern countries showing higher rates of IT-HPV detection. These findings may reflect differences in HPV detection prevalence across regions, as well as potential variability in epidemiological factors, study populations, and detection methods, rather than supporting a definitive etiological role of HPV in PCa. Further research is needed to clarify the relationship between HPV presence, specific mutational patterns, and treatment outcomes.

## Figures and Tables

**Table 1:** Clinical and molecular characteristics of HPV DNA-positive patients in the Swiss cohort. GS, Gleason score; iPSA, initial PSA; OS, overall survival; NA, not available.

	Age at the Diagnosis	Gleason Score	iPSA (mPCa)	mPCa De Novo/Metachronous	Volume Disease	Treatment	OS	OS Only for Metastatic Disease	HPV Type	Molecular Profile on NGS
Patient 1	87 y	9 (4 + 4)	264	De novo	High	ADT + ARPI	34.5 mo	34.5 mo	66	TP53 C275FfsTer71
Patient 2	56 y	10 (5 + 5)	127	Metachronous	Low	Surgery; Ciproteron; Radiotherapy; ADT + ARPI; Docetaxel	329.5 mo	92.3 mo	16	CDKN1B; SPOP D130H
Patient 3	72 y	8 (4 + 4)	27.07	Metachronous	High	ADT	237.7 mo	43.2 mo	6	NA
Patient 4	55 y	7 (4 + 3)	123	Metachronous	Low	Surgery; Radiotherapy; ADT + ARPI; Docetaxel; Xaluritamig	155 mo	90.6 mo (update)	16	PIK3CA p.H1047R; POLE p.lle1871Val SNV
Patient 5	74 y	8 (4 + 4)	2.1	Metachronous	High	Radiotherapy; ADT + ARPI	78 mo	11.6 mo	16	NA
Patient 6	58 y	NA	670	De novo	High	Docetaxel; ADT + ARPI; Radium-223 Cabazitaxel Lu-PSMA Carboplatinum	52.7 mo	52.7 mo	16	SPOP p.W131C; FANCA pSer447Leu

## Data Availability

The data generated and analyzed during the current study are not publicly available because they contain potentially identifiable clinical and molecular information and are subject to ethical and data protection restrictions. De-identified data may be made available by the corresponding author upon reasonable request, subject to approval by the competent ethics committee and the relevant institutional data governance procedures.

## Supplementary Materials

The following supporting information can be downloaded at: https://www.mdpi.com/article/10.3390/ijms27177831/s1.

## Abbreviations

The following abbreviations are used in this manuscript:

ADT	Androgen deprivation therapy
ARPI	Androgen-receptor pathway inhibitor
FFPE	Formalin-fixed paraffin-embedded
GS	Gleason Score
HPV	Human papillomavirus
IDISI	Institute of Integrated Diagnostics of Italian Switzerland
IOSI	Oncology Institute of Southern Switzerland
IT	Intratumoral
LA-PCa	Locally advanced prostate cancer
mHSPC	Metastatic hormone-sensitive prostate cancer
mPCa	Metastatic prostate cancer
NGS	Next-generation sequencing
PCa	Prostate cancer
PFS	Progression-free survival
PSA	prostate-specific antigen
OS	Overall Survival
TURP	Transurethral Resection of the Prostate.
