# Supplementary material for: Intratumoral Human Papillomavirus in Advanced Prostate Cancer: Systematic Review, Meta-Analysis, and Real-World Evidence from the Largest Cohort to Date
Source: Int J Mol Sci. 2026 Sep 1;27(17):7831. doi: 10.3390/ijms27177831 (PMC13566354; doi:10.3390/ijms27177831)
Supplement: Supplementary file 1 [file ijms-27-07831-s001.zip › ijms-4480611-supplementary.pdf]

SUPPLEMENTARY MATERIALS

Intratumoral Human Papillomavirus in Advanced Prostate Cancer: Systematic Review, Meta-Analysis, and Real-World Evidence from the Largest Cohort to Date

Daniele Brenna [1,†], Marialuisa Puglisi [2,*,†], Samantha Epistolio [3], Jessica Barizzi [3], Martino Pedrani [2], Giuseppe Salfi [2], Giovanna Pecoraro [2], Fabio Turco [2], Luigi Tortola [2], Salvatore Cozzi [1], Thomas Zilli [1,4,5], Gianmarco Leone [2], Ursula Vogl [2], Silke Gillessen [2,4], Milo Frattini [3,‡] and Ricardo Pereira Mestre [2,4,‡]

[1] Radiation Oncology, Oncology Institute of Southern Switzerland, Ente Ospedaliero Cantonale, CH 6500 Bellinzona, Switzerland; daniele.brenna@eoc.ch (D.B.); salvatore.cozzi@eoc.ch (S.C.); thomas.zilli@eoc.ch (T.Z.)

[2] Medical Oncology, Oncology Institute of Southern Switzerland (IOSI), Ente Ospedaliero Cantonale (EOC), CH 6500 Bellinzona, Switzerland; martino.pedrani@eoc.ch (M.P.); giuseppe.salfi@eoc.ch (G.S.); giovanna.pecoraro@eoc.ch (G.P.); fabio.turco@eoc.ch (F.T.); luigi.tortola@eoc.ch (L.T.); gianmarco.leone@eoc.ch (G.L.); ursula.vogl@eoc.ch (U.V.); silke.gillessensommer@eoc.ch (S.G.); ricardo.pereiramestre@eoc.ch (R.P.M.)

[3] Laboratory of Genetics and Molecular Pathology, Institute of Pathology, Ente Ospedaliero Cantonale (EOC), CH 6600 Locarno, Switzerland; samantha.epistolio@eoc.ch (S.E.); jessica.barizzi@eoc.ch (J.B.); milo.frattini@eoc.ch (M.F.)

[4] Faculty of Biomedical Sciences, Università della Svizzera Italiana, CH 6900 Lugano, Switzerland

[5] Faculty of Medicine, Geneva University, CH 1211 Geneva, Switzerland

[*] Correspondence: marialuisa.puglisi@eoc.ch

[†] These authors contributed equally to this work.

[‡] These authors also contributed equally to this work.

S1. Literature search strategy

A systematic literature search was conducted to identify all relevant studies. The search strategy included electronic database searches and manual screening of reference lists

<u>Electronic Database Search</u>

Scopus:

(("prostate cancer" OR "prostatic neoplasm*") AND ("hpv" or "human papillomavirus"))

In Scopus, the research was limited to the title, abstract, and keyword fields.

PubMed:

(("prostate cancer" OR "prostatic neoplasms") AND ("hpv" or "human papillomavirus" or "papillomavirus infections"))

In PubMed, the research included terms in the title and abstract fields, as well as the MeSH (Medical Subject Headings) terms.

Web of Science:

(("prostate cancer" OR "prostatic neoplasm*") AND ("hpv" OR "human papillomavirus"))

In Web of Science, the research was limited to the Topic field (which includes title, abstract, and keywords).

<u>Ancillary search strategies</u>

In addition to the electronic search, the reference lists of all included articles and previously published relevant systematic reviews and meta-analyses were manually screened to identify any potentially eligible studies not captured by the database search.

S2. Exclusion criteria

Exclusion criteria for the systematic review included: (a) articles not relevant to the field of interest; (b) systematic reviews and meta-analyses, editorials, comments, letters to the editor, case reports within the field of interest; (c) in cases of potential data overlap among eligible articles, only the most comprehensive article was included in the meta-analysis, while both were included in the systematic review (d) lack of sufficient data on disease staging (e) incorrect patient population (e.g. cohort exclusively comprising non-metastatic disease) (f) required data not reported for the metastatic subgroup (e.g. absence of relative number of metastatic cases) (g) HPV assessed as a marker in benign, not malignant, tissue (h) incorrect method of HPV detection (e.g. status based on serological or other indirect evidence, not on direct intratumoral analysis)

S3. Methods

All statistical analyses for the EOC real-world cohort were performed using Python (version 3.11), relying on the pandas library (version 2.2.2) and the numpy library (version 1.26.4).

The primary dataset was loaded into a pandas DataFrame for processing and analysis.

Descriptive statistics for the entire retrospective cohort were generated using functions from the pandas and numpy libraries.

Continuous variables were summarized by calculating the mean, median, interquartile range (IQR) and the 95% confidence interval (CI) of the mean. Categorical variables were summarized as frequencies and percentages of the total cohort, or of the subset of patients for whom the data was evaluable.

As per the final analysis presented, no formal statistical comparisons were performed between the HPV-positive and HPV-negative subgroups due to the small number of positive cases, which would render such comparisons statistically unreliable.

The summarized counts for genomic alterations (e.g., 'TP53, altered', 'PTEN/PI3K pathway, altered') presented in Supplementary Materials (Table S2), were derived by consolidating specific pathogenic or likely pathogenic variants identified in the NGS reports.

Table S1

Study (Year)	Region	N. of advanced patients	HPV Prevalence in advanced cases (%)	Genotypes detected	Genotypes detected in advanced group	Oncogenic risk category	Association with Gleason Score	Specimen site	HPV detection method	Other molecular mechanisms investigated
**Anwar et al. (1992) [21]**	Japan	47	26 (55.3%)	HPV-16, 18, 33	HPV-16 (n=4), 18 (n=12), 33 (n=3)	HR	Positive (p<0.005)	Primary tumor & metastatic sites	PCR & DNA Hybridization	ras gene mutations (positive association). HPV-18 often found in patients with bone metastases. Association between tumor stage and HPV positivity
**Tu et al. (1994) [29]**	USA	17	1 (5.9%)	HPV-18	HPV-18 (n=1)	HR	Not reported	Metastatic pelvic lymph nodes	PCR & Southern Blot	None
**Suzuki et al. (1996) [22]**	Japan	22	3 (13.6%)	HPV-16	HPV-16 (n=3)	HR	Not reported	Primary and metastatic sites (autopsy)	PCR (L1) & RFLP	p53 mutations (inverse correlation with HPV positivity)
**Anderson et al. (1997) [30]**	England	3	0%	None	None	Not applicable	Not applicable	Primary tumor (TURP)	PCR + primers specific to HPV-16 E6 and E2	None
**Serth et al. (1999) [31]**	Germany	20	Not reported	HPV-16	Not reported	Not applicable	Positive (p val not reported)	Primary tumor (surgery)	quantitative competitive PCR	HPV-16 DNA copy number
**Zambrano et al. (2002) [32]**	USA	5	2 (40%)	HPV-16, 18, 33, 58	HPV 58 (n=1), HPV-33 (n=1)	HR	Not reported	Primary tumor (surgery)	PCR with consesus primers (MY09/11 and GP5+/6+)	BKV and JCV
**Balis et al. (2007) [33]**	Greece	14	Not reported	**Not** HPV-11,16,18,33)	Not reported	Not applicable	None	Primary tumor (surgery)	PCR with consesus primers (GP5+/6+)	BKV and JCV
**Silvestre et al. (2009) [34]**	Brazil	28[1]	0	None	None	Not applicable	Not applicable	Primary tumor (surgery)	PCR + LINEAR ARRAY	None
**Tachezy et al (2012) [35]**	Czech Republic	17	Not reported	HPV-42	Not reported	LR	Not reported	Primary tumor(surgery)	Nested PCR (MY09/11 followed by GP5+/6+)	Serology
**Salehi & Hadavi (2012) [23]**	Iran	68	3 (4,4%)	Not reported	Not reported	Not applicable	None	Primary tumor (biopsy)	PCR (consensus primers)	TP53 codon 72 polymorphism analysis
**Ghasemian et al. (2013) [24]**	Iran	6	2 (33.3%)	Not reported	Not reported	Not applicable	Positive (higher mean Gleason)	Primary tumor (TURP/surgery)	Nested PCR	None
**Michopoulou et al. (2014) [36]**	Greece	11	2 (18,1%)	HPV-18, 16, 31	HPV-18 (n=1)	HR	None	Primary tumor (surgery)	Real-time PCR + LINEAR ARRAY	TP53 codon 72 polymorphism (no association)
**Yow et al. (2014) [37]**	Australia	49 ('aggressive disease')[2]	0	None	None	Not applicable	Not applicable	Primary tumor (TURP/surgery)	PCR (HR KIT) + ELISA	Panel of 10 microorganisms
**Singh et al. (2015) [25]**	India	23	17 (73.9%)	HPV-16, 18, 6	HPV-6 (n=1), HPV-16 (n=13), HPV-18 (n=3)	HR, only one LR	Positive (p=0.003)	Primary tumor (biopsy)	PCR (Consensus + type specific)	None
**Araujo-Neto et al. (2016) [38]**	Brazil	76[1]	0%	None	None	Not applicable	Not applicable	Primary tumor (surgery)	Nested PCR	None
**Nahand et al. (2020) [10]**	Iran	33	15 (45,4%)	HPV-16, 18, 33	Not reported	HR	Not reported	Primary tumor (surgery)	PCR + INNO-LIPA kit	Inflammation, Angiogenesis, Apoptosis (p53/Rb), Integration Status
**Fatemipour et al. (2021) [26]**	Iran	43*	20 (46,5%)	HPV-16, 18, 33	Not reported	HR	Not reported, but integration status investigated stratified by stage	Primary tumor (surgery)	PCR + INNO-LIPA kit	Anoikis Resistance, Epithelial-Mesenchymal Transition (EMT), integration status
**Nahand et al. (2021) [7]**	Iran	40	Not reported	HPV-16, 18, 33, 6	Not reported	HR and LR	Not reported	Primary tumor (surgery)	PCR + INNO-LIPA kit	HPV/EBV Co-infection, Inflammation, Anoikis, Integration Status
**Khatami et al. (2022) [27]**	Iran	44	17 (38,6%)	HPV-16, 18, 33, 6, 11	Not reported	HR and LR	Not reported	Primary tumor (surgery)	PCR (consensus primers)	miRNA profile, p53/Rb, Apoptosis markers, Integration Status
**Nellessen et al. (2023) [39]**	Germany	52[1]	2 (3,8%)	HPV-6, 11, 16, 18, 54, 91	Not reported	HR and LR	None	Primary tumor (surgery)	PCR + LCD-array hybridization	Sociodemographic factors
**Andersen et al. (2024) [40]**	Denmark & France	88	0%	None	None	Not applicable	Not applicable	Primary tumor (surgery)	Deep panel sequencing - NGS	Broad viral screening including 18 different families

Study (Year)	Region	N. of advanced patients	HPV Prevalence in advanced cases (%)	Genotypes detected	Genotypes detected in advanced group	Oncogenic risk category	Association with Gleason Score	Specimen site	HPV detection method	Other molecular mechanisms investigated
**Lang et al. (2024) [28]**	China	26[1]	4 (15,4%)	HPV 16, 18 and others	Not reported	HR	Not applicable	Primary tumor (surgery)	Capture sequencing - NGS	Genomic mutational profile (WES), CNV, Integration status
**Pina-Vaz et al. (2025) [5]**	Portugal	20[1]	Not reported[3]	Not reported	Not reported	Not applicable	Not reported	Primary tumor (biopsy)	PCR	Panel of 7 STIs

[1]Note: Only T3 and T4 cases were considered when it was not possible to further stratify between N+, M+ and T stages
[2]Note: Aggressive disease was defined as Gleason score ≥7,<br>poorly-differentiated, including tumours staged at T4,<br>N+(lymph node positive), or M+(distant metastases). However, the prevalence of HPV was 0% in this class. Thus they were included in the count.<br>regardless of their Gleason score or grade of differentiation.
[3]Note: The Pina-Vaz et al. (2025) [5] study identifies the metastatic subgroup but does not report HPV prevalence specifically for it.
*72 patients are reported in the table while in the 'Methods' section 58 patients are said to be enrolled (correct: 72)

Legend:

RFLP: Restriction Fragment Length Polymorphism

LCD: Liquid Crystal Detection

Study (Year)	Region	N. of metastatic patients	HPV Prevalence in metastatic (%)	Setting (mHSPC/ mCRPC)[1]	Genotypes detected	Genotypes detected in metastatic subgroup	Oncogenic risk category	Association with Gleason Score	Specimen site	HPV detection method	Other molecular mechanisms/viruses investigated
**Anwar et al. (1992) [21]**	Japan	37	20 (54.1%)	Not reported	HPV-16, 18, 33	HPV-16 (n=4), 18 (n=12), 33 (n=3)	HR	Positive (p<0.005)	Primary tumor & metastatic sites	PCR & DNA Hybridization	ras gene mutations (positive association). HPV-18 often found in patients with bone metastases. Association between tumor stage and HPV positivity

**Study (Year)**	**Region**	**N. of metastatic patients**	**HPV Prevalence in metastatic (%)**	**Setting (mHSPC/ mCRPC)[1]**	**Genotypes detected**	**Genotypes detected in metastatic subgroup**	**Oncogenic risk category**	**Association with Gleason Score**	**Specimen site**	**HPV detection method**	**Other molecular mechanisms/viruses investigated**
**Tu et al. (1994) [29]**	USA	17	1 (5.9%)	Not reported	HPV-18	HPV-18 (n=1)	HR	Not reported	Metastatic pelvic lymph nodes	PCR & Southern Blot	None
**Suzuki et al. (1996) [22]**	Japan	22	3 (13.6%)	mCRPC[1]	HPV-16	HPV-16 (n=3)	HR	Not reported	Primary and metastatic sites (autopsy)	PCR (L1) & RFLP	p53 mutations (inverse correlation with HPV positivity)
**Silvestre et al. (2009) [34]**	Brazil	31[2]	0%	Not reported	None	None	Not applicable	Not applicable	Primary tumor (surgery)	PCR + LINEAR ARRAY	None
**Salehi & Hadavi (2012) [23]**	Iran	68 ("Advanced Stage")[3]	Not reported (4,4% in the whole cohort)	Not reported	Not reported	Not reported	Not applicable	None	Primary tumor (biopsy)	PCR (consensus primers)	TP53 codon 72 polymorphism analysis
**Ghasemian et al. (2013) [24]**	Iran	6	2 (33.3%)	Not reported	Not reported	Not reported	Not applicable	Positive (higher mean Gleason)	Primary tumor (TURP/surgery)	Nested PCR	None
**Michopoulou et al. (2014) [36]**	Greece	5	2 (40%)	Not reported	HPV-18, 16, 31	HPV-18 (n=1)	HR	None	Primary tumor (surgery)	Real-time PCR + LINEAR ARRAY	TP53 codon 72 polymorphism (no association)
**Yow et al. (2014) [37]**	Australia	49 ('aggressive disease', metastatic patients included)[4]	Not reported (0% in the 'aggressive' disease class)	Not reported	None	None	Not applicable	Not applicable	Primary tumor (TURP/surgery)	PCR (HR KIT) + ELISA	Panel of 10 microorganisms
**Singh et al. (2015) [25]**	India	23 ('advanced', including stage III and stage IV patients)	Not reported (73.9% in the 'advanced' class)	Not reported	HPV-16, 18, 6	Reported for stage III+IV	HR and LR	Positive (p=0.003)	Primary tumor (biopsy)	PCR (Consensus + type specific)	None
**Araujo-Neto et al. (2016) [38]**	Brazil	19[2]	0%	Not reported	None	None	Not applicable	Not applicable	Primary tumor (surgery)	Nested PCR	None
**Nahand et al. (2020) [10]**	Iran	16	8 (50%)	Not reported	HPV-16, 18, 33	Not reported	HR	Not reported	Primary tumor (surgery)	PCR + INNO-LIPA kit	Inflammation, Angiogenesis, Apoptosis (p53/Rb), Integration Status
**Fatemipour et al. (2021) [26]**	Iran	22*	11 (50%)	Not reported	HPV-16, 18, 33	Not reported	HR	Not reported, but integration status investigated stratified by stage	Primary tumor (surgery)	PCR + INNO-LIPA kit	Anoikis Resistance, Epithelial-Mesenchymal Transition (EMT), integration status
**Nahand et al. (2021) [7]**	Iran	22	Not reported	Not reported	HPV-16, 18, 33, 6	Not reported	HR	Not reported	Primary tumor (surgery)	PCR + INNO-LIPA kit	HPV/EBV Co-infection, Inflammation, Anoikis, Integration Status
**Khatami et al. (2022) [27]**	Iran	22	9 (41%)	Not reported	HPV-16, 18, 33, 6, 11	Nor reported	HR and LR	Not reported	Primary tumor (surgery)	PCR (consensus primers)	miRNA profile, p53/Rb, Apoptosis markers, Integration Status
**Nellessen et al. (2023) [39]**	Germany	13	0%	Not reported	HPV-6, 11, 16, 18, 54, 91	Not reported	HR and LR	None	Primary tumor (surgery)	PCR + LCD-array hybridization	None
**Lang et al. (2024) [28]**	China	6[2]	2 (33%)	Not reported	HPV 16, 18 and others	Not reported	HR and LR	Not applicable	Primary tumor (surgery)	Capture sequencing	Genomic mutational profile (WES), CNV, Integration status

Study (Year)	Region	N. of metastatic patients	HPV Prevalence in metastatic (%)	Setting (mHSPC/ mCRPC)[1]	Genotypes detected	Genotypes detected in metastatic subgroup	Oncogenic risk category	Association with Gleason Score	Specimen site	HPV detection method	Other molecular mechanisms/viruses investigated
**Pina-Vaz et al. (2025) [5]**	Portugal	51[2]	Not reported[5]	Not reported	Not reported	Not reported	Not applicable	Not reported	Primary tumor (biopsy)	PCR	Panel of 7 STIs

[1]*Note: The distinction between mHSPC and mCRPC is extremely rare in the analyzed literature. Only the study by Suzuki et al. (1996) [22] clearly specifies "endocrine therapy-resistant," classifying the patients as mCRPC.*
[2]*Note: only N+ and M+ cases were considered where it was impossible to stratify between N+, M+ and T stages*
[3]*Note: all the cases in the study were defined as 'advanced' with no further specification*
[4]*Note: Aggressive disease was defined as Gleason score ≥7, poorly-differentiated, including tumours staged at T4, N+(lymph node positive), or M+(distant metastases). However, the prevalence of HPV was 0% in this class. Thus they were included in the count regardless of their Gleason score or grade of differentiation.*
[5]Note: The Pina-Vaz et al. (2025) [5] study identifies the metastatic subgroup but does not report HPV prevalence specifically for it.
**72 patients are reported in the table while in the 'Methods' section 58 patients are said to be enrolled (correct: 72)*

Table S1: Descriptive tables of the included studies. Above, all the locally advanced prostate cancer studies. Below, all metastatic prostate cancer studies

HPV genotypes were categorized according to oncogenic risk as high-risk (HR-HPV) or low-risk/non-high-risk (LR-HPV) types. When genotype information was reported for the overall study population but could not be specifically attributed to the advanced-stage subgroup, no subgroup-specific risk classification was assigned.

Figure S1A

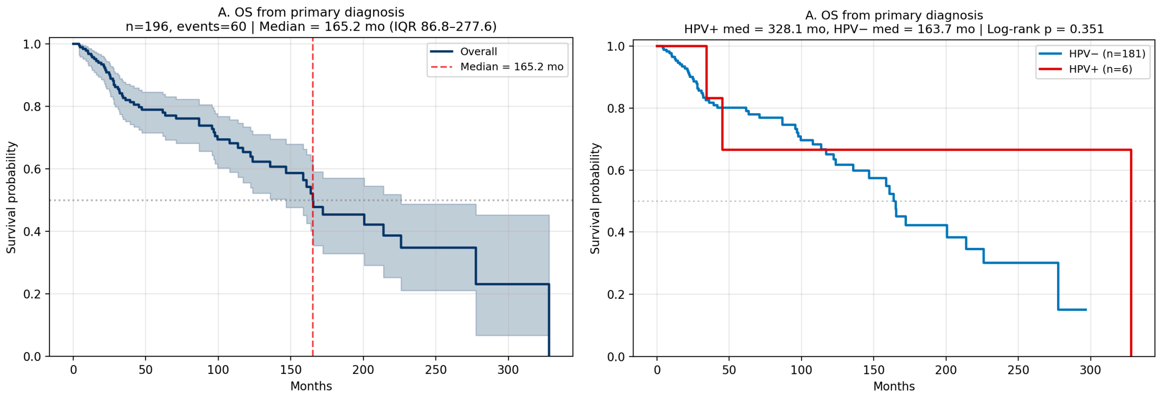

Figure S1A. Kaplan–Meier overall survival (OS) from primary diagnosis in the metastatic PCa cohort. Right panel: OS in the overall population. Left panel: OS stratified by HPV status; no statistically significant difference was observed between groups.

Figure S1B

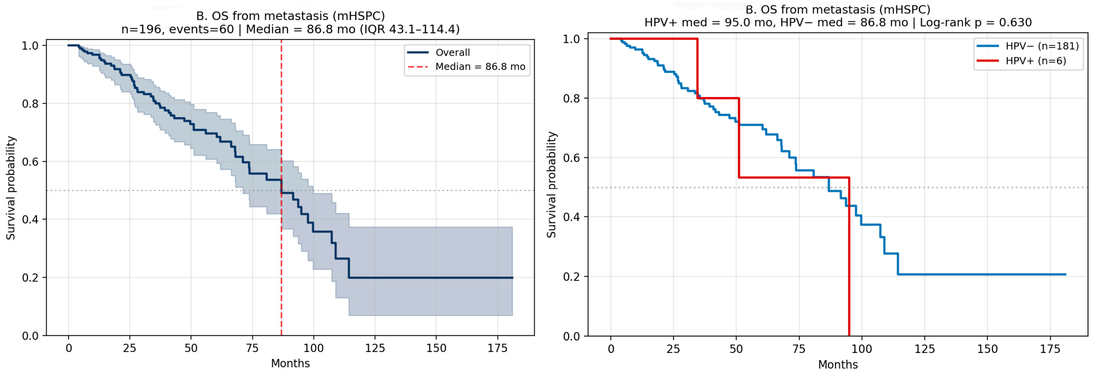

Figure S1B. Kaplan–Meier overall survival (OS) from mHSPC diagnosis in the metastatic PCa cohort. Right panel: OS in the overall population. Left panel: OS stratified by HPV status; no statistically significant difference was observed between groups.

Figure S1C

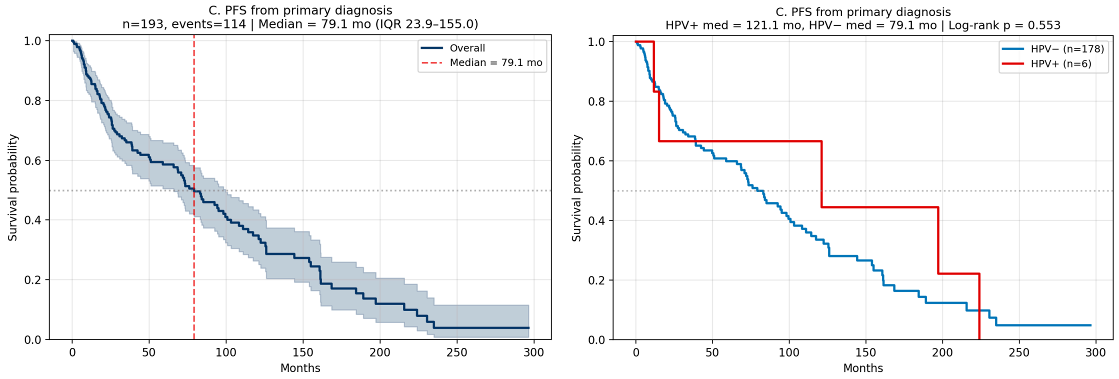

Figure S1C. Kaplan–Meier progression-free survival (PFS) from primary diagnosis in the metastatic PCa cohort. Right panel: PFS in the overall population. Left panel: PFS stratified by HPV status; no statistically significant difference was observed between groups.

Figure S1D

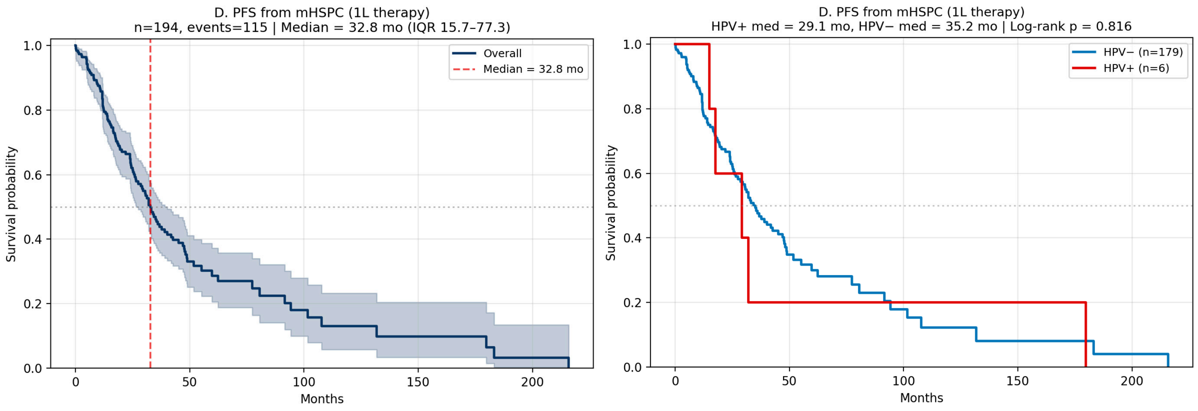

Figure S1D. Kaplan–Meier progression-free survival (PFS) from initiation of first-line mHSPC therapy in the metastatic PCa cohort. Right panel: PFS in the overall population. Left panel: PFS stratified by HPV status; no statistically significant difference was observed between groups.

**Characteristic**	**Category / Statistic**	**Value**
**Demographic and Clinical Characteristics**		n (%) or Mean/Median
Age at diagnosis	Mean (95% CI)	69.91 (68.56 - 71.26)
	Median [IQR]	69.67 [62.72 - 76.23]
PSA at metastatic diagnosis (ng/mL)	Mean (95% CI)	169.47 (84.42 - 254.53)
	Median [IQR]	29.35 [9.97 - 94.12]
Median follow-up time from primary diagnosis (months)	Mean (95% CI)	75.70 (65.91 - 85.48)
	Median [IQR]	50.13 [22.82 - 113.44]
OS from primary diagnosis (Kaplan–Meier median, months)	Median	165.2
	IQR	86.8 - 277.6; n=196, 60 deaths
Median follow-up time from metastatic diagnosis (months	Mean (95% CI)	42.9 (38.4 - 47.4).
	Median [IQR]	37.4 [16.5 - 61.1]
OS from metastatic diagnosis (months)	Median	86.8
	IQR	43.1 - 114.4 n= 196, 60 deaths
Median time on first-line mHSPC therapy (descriptive, months)	Median [IQR]	23.20 [11.9 – 41.8]
PFS from initiation of first-line mHSPC therapy (Kaplan–Meier median, months)	Mean	*32.8*
	IQR	15.7 - 77.3; n=194, 115 PFS events; n = 194, 115 PFS events
Disease Status at Diagnosis		196 (100%)
	De novo	120 (61.2%)
	Metachronous	75 (38.3%)
	Locally advanced	1 (0.5%)
Disease Volume (CHAARTED)		196 (100%)
	High Volume	109 (55.6%)
	Low Volume	87 (44.4%)
First-Line Treatment (mHSPC)		196 (100%)
	ADT + ARTA	93 (47.4%)
	ADT alone	72 (36.7%)
	ADT + Chemotherapy	15 (7.7%)

Characteristic	Category / Statistic	Value
	ADT + ARTA + Chemotherapy	13 (6.6%)
	Other	3 (1.5%)
Biopsy Site for NGS		196 (100%)
	Prostate	151 (77.0%)
	Bone	11 (5.6%)
	Lymph Nodes	11 (5.6%)
	Liver	10 (5.1%)
	Soft Tissue	3 (1.5%)
	Blood	2 (1.0%)
	Lung	2 (1.0%)
	Mixed (Prostate/Liver, Prostate/Lymph Node)	2 (1.0%)
	Other (Brain, Pleural, Peritoneal, Adrenal)	4 (2.0%)
HPV Status		196 (100%)
	Negative	181 (92.3%)
	Positive, variant 16 (high risk)	4 (2.0%)
	Positive, variant 66 (high risk)	1 (0.5%)
	Positive, variant 6 (low risk)	1 (0.5%)
	Not Evaluable	9 (4.6%)
Microsatellite Instability (MSI) Status	Assessed in 189 patients	
	Stable (MSS)	179 (94.7%)
	High Instability (MSI-H)	7 (3.7%)
	Low Instability (MSI-L)	3 (1.6%)
	Not Evaluable	7 patients
**Genomic Alterations**		n (% of assessed patients)
TP53, altered	Assessed in 178	50 (28.1%)[1]
PTEN/PI3K pathway, altered	Assessed in 178	22 (12.4%)[2]
SPOP, altered	Assessed in 178	17 (9.6%)[3]

Characteristic	Category / Statistic	Value
DDR (other), altered	Assessed in 178	11 (6.2%)[4]
BRCA1/2, altered	Assessed in 182	10 (5.5%)[5]
MYC, altered	Assessed in 178	8 (4.5%)[6]
AR, altered	Assessed in 178	8 (4.5%)[7]
CTNNB1, altered	Assessed in 177	8 (4.5%)[8]
CDKN1B, altered	Assessed in 177	6 (3.4%)[9]
AKT, altered	Assessed in 177	5 (2.8%)[10]
FGFR, altered	Assessed in 177	4 (2.3%)[11]
RB1, altered	Assessed in 178	3 (1.7%)[12]
NOTCH, altered	Assessed in 177	3 (1.7%)[13]
NF1, altered	Assessed in 177	3 (1.7%)[14]
CCNE1, altered	Assessed in 177	3 (1.7%)[15]
RAS (KRAS), altered	Assessed in 178	2 (1.1%)[16]
MSH2, altered	Assessed in 177	2 (1.1%)[17]
FBXW7, altered	Assessed in 176	2 (1.1%)[18]
ERG translocation, altered	Assessed in 178	1 (0.6%)[19]
Other genes (1 case each)	XPO1, SMAD4, IDH1, ARID1A, MAP2K1, CHD1, IGF, etc.	See note [20]

Mutated Genes	Notes
[1] **TP53 (n=50)**	Includes p.R175H (2), C275FfsTer71, p.I255del, p.Pro190Leu, p.S241F, p.Y126H, H193Y, p.L194F, R282W, p.R248W, c.920-2A>G, p.D281H, p.R248W and p.R273H, V157F, p.S166*, p.T284Qfs61, p.Y126C, p.Y220C, p.S183, p.R273H, p.R110P, p.R306Ter, p.E343Wfs22, p.P152L and p.W91Cfs32, R273C, c.560-2A>T, p.S306*, p.H193R, p.P250L, p.S261Rfs2, p.R282W, p.S94Ter, p.R196, p.T125K, p.H179L, p.Y220N, V143E, H193P, L257Q, p.I251S, p.V147G, p.K291RfsTer54, p.H214R, p.Y126C, M133K, p.E294*, and other generic descriptions ("TP53 mutation").
[2] **PTEN/PI3K pathway (n=22)**	Includes alterations in PTEN (R55Sfs39, c.206_209+2del, p.R130Ter, p.L247, p.R173C, p.Y68Ifs6 and p.N329Kfs14, p.T319Ter, p.T319, p.R173C and p.P246L, Y16*, E40DfsTer13), PIK3CA (p.H1047R (2), p.Q546H, p.P449_E453del, p.E545A, p.E110, p.E545K, p.Q546R), PIK3R1 (p.R631Ter, p.Q579Hfs*3), PIK3CB (copy number gain), AKT2 (copy number gain).
[3] **SPOP (n=17)**	Includes p.F133L (2), p.W131C (2), p.W131R, D130H, p.F102V, p.F133V, p.F125V, p.W131G, p.K129E, p.F102C, and other variants of F133, F125, W131.
[4] **DDR (other genes) (n=11)**	Includes alterations in FANCD2 (p.E1435Sfs10), NBN (p.Q681), ATRX (p.R37*), PALB2 (somatic heterozygous), RAD50 (p.L31Pfs17), CDK12 (p.R388Ifs45, p.K852Ifs5), BAP1 (p.Q36Rfs36), ATM (p.Q1225*, Q2733fs18), RAD51B (p.R8).
[5] **BRCA1/2 (n=10)**	Includes alterations in BRCA1 (p.S282Yfs15) and BRCA2 (L3125H, G1338fs36, p.I605Yfs9, T3199S, c.67+1G>A, c.7618-1G>A, p.N886KfsTer2, p.Y2215LfsTer10) and BRCA1/2 (p.L718Qfs7).
[6] **MYC (n=8)**	Copy number gain / amplification (with copy numbers ranging from 6 to 25).
[7] **AR (n=8)**	Copy number gain / amplification (with copy numbers ranging from 10 to 21.3) and one case of exon 5-7 deletion.
[8] **CTNNB1 (n=8)**	Includes p.K335I, T41I, p.T41I, p.S33C, p.S37C, p.D32Y, S37C, H36P.
[9] **CDKN1B (n=6)**	Includes p.N180Mfs45, p.K100Sfs13, p.P137Rfs8, p.S175Qfs50, one VUS, and one case of slight copy number gain.
[10] **AKT (n=5)**	Includes alterations in AKT1 (p.E17K (3), p.L52R) and AKT3 amplification.
[11] **FGFR (n=4)**	Copy number gain of FGFR1, and amplifications/copy number gains of FGF19, FGF3, FGF4.
[12] **RB1 (n=3)**	Includes p.R455*, p.A74Efs*4, c2326-8_2330del.
[13] **NOTCH (n=3)**	Includes alterations in NOTCH1 (copy number gain) and NOTCH2 (p.E2020*, P2288Efs*21).
[14] **NF1 (n=3)**	Includes p.N292VfsTer17 and p.K1588NfsTer34, p.R461*, and one "NF1 mutation".
[15] **CCNE1 (n=3)**	Copy number gain / amplification.
[16] **RAS (KRAS) (n=2)**	Includes p.G12V and Q61L.
[17] **MSH2 (n=2)**	Includes p.E876* and p.A604GfsTer40.
[18] **FBXW7 (n=2)**	Includes p.R505G and p.R505L.
[19] **ERG translocation (n=1)**	TMPRSS2-ERG fusion.
[20] **Other genes (1 case each)**	XPO1 (p.E571K), SMAD4 (mutation), IDH1 (p.R132H), ARID1A (mutation), MAP2K1 (p.E102_I103del), CHD1 (c.1320+1G>A), IGF1R (copy number gain), PDGFR (copy number gain), KIT (copy number gain), KDR (copy number gain), GNAS (p.R201C), FLT3 (copy number gain), ERBB3 (p.V104M), PTCH1 (copy number gain), CCND1 (copy number gain), CAMK1D - PHF1 (fusion), TET2 (E433*). EZH2, DNMT3A, APC genes had no alterations in the assessed cases.

Table S2: Summary tables of Swiss cohort in 196 patients

Results S1. Risk of bias assessment

Studies appraised with the JBI critical appraisal criteria

The methodological quality was found to be variable, with two studies demonstrating high rigor and three studies presenting notable concerns in specific domains. Specifically, the studies conducted by Araujo Neto et al. [38] and Andersen et al. [40] were judged to be at a low risk of bias across all assessed items.

In contrast, the remaining three studies presented a mixed risk profile. The study by Yow et al. [37] was judged to be at high risk of bias in the domains of consecutive and complete inclusion of the participants. These same two domains were rated as having an unclear risk of bias in the studies by Balis et al. [33] and Tu et al. [29] Furthermore, also clear reporting of the patients' demographics and clinical information were rated as unclear risk for Balis et al. [33] Tu et al. [29] was evaluated at unclear risk of bias also for the presenting sites' item.

Figure S2: risk of bias according to the JBI critical appraisal tool for case series included in the review. Traffic light graph (above) and summary plot (below)

Studies appraised with the NOS scale

Advanced Disease Cohort

Eighteen studies were evaluated as the advanced-stage disease cohort. The risk of bias assessment for these studies is detailed and summarized in Figure S3. A high risk of bias was predominantly identified in the Comparability domain. Additionally, a notable number of studies raised some concerns regarding the Selection item. In contrast, the domain concerning the Outcome and Exposure was consistently judged to be at low risk of bias.

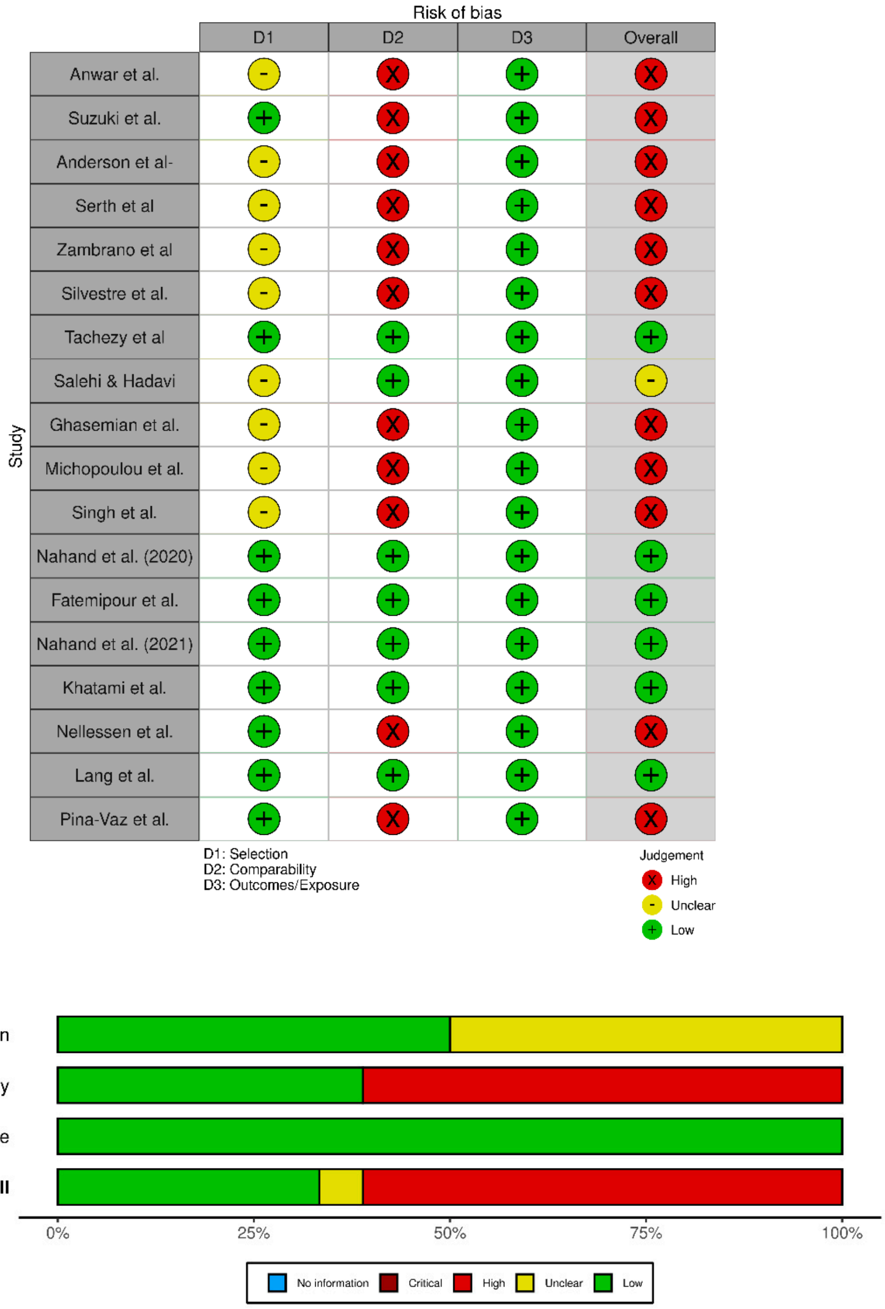

Figure S3: risk of bias according to the NOS scale for case control studies focused on the advanced disease cohorts included in the review. Traffic light graph (above) and summary plot (below)

<u>Metastatic Disease Cohort</u>

The fourteen studies that specifically included patients with metastatic disease were analyzed as a distinct subgroup. The assessment results for this cohort are presented in Figure S4. Similar to the broader advanced cohort, the most significant methodological issue was the Comparability of cohorts, which was rated at high risk of bias for a majority of the studies. Some concerns were also frequently identified for the Selection domains. The domains related to the Outcomes and Exposure were generally rated at low risk of bias.

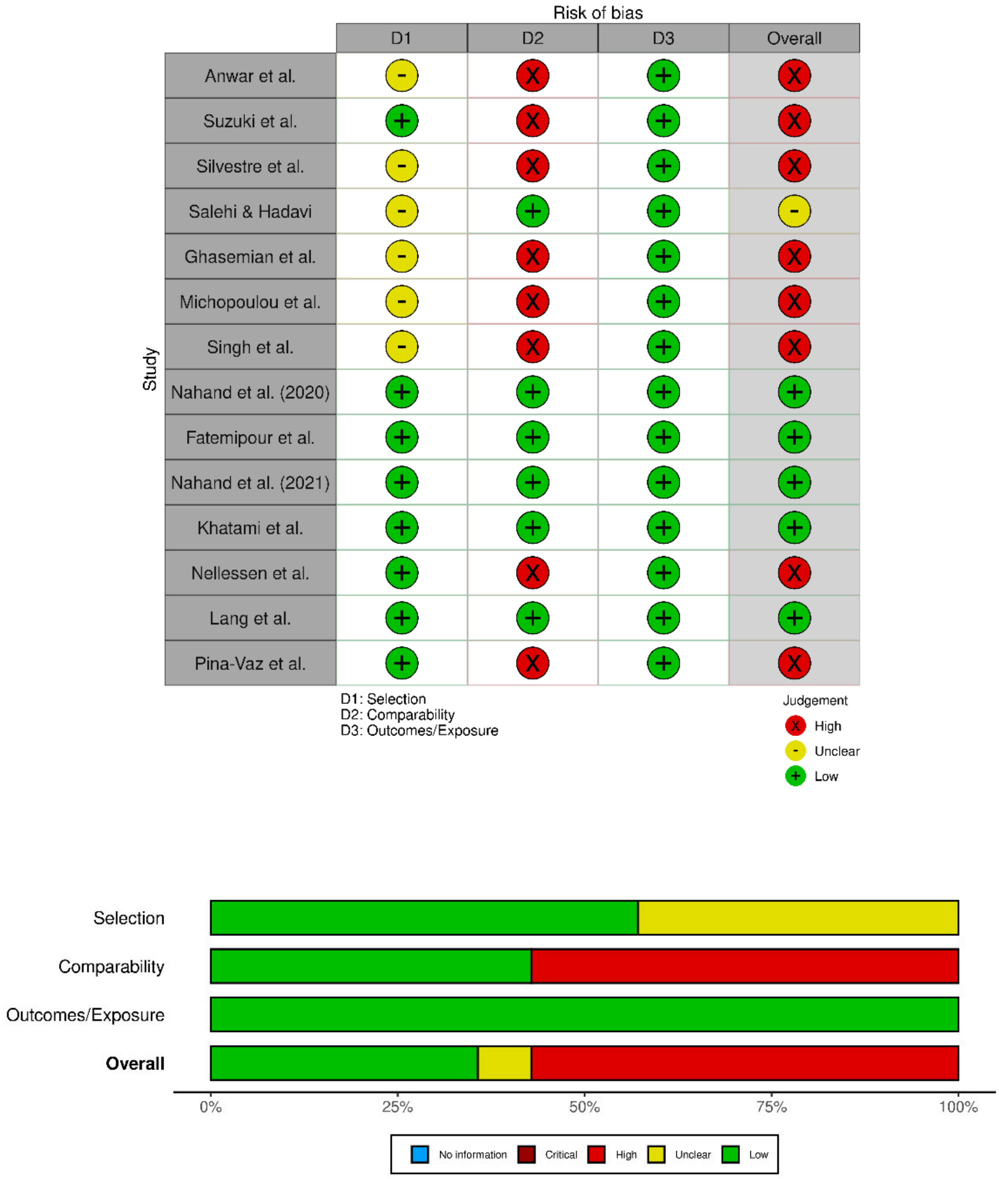

Figure S4: risk of bias according to the NOS scale for case control studies focused on the metastatic cohorts included in the review. Traffic light graph (above) and summary plot (below)

Results S2. Assessment of Publication Bias

Publication bias was assessed visually using funnel plots and formally tested using Egger's linear regression test for both primary meta-analyses.

Figure S5 presents the funnel plot for the 'Advanced Stage' meta-analysis (k=18 studies). Visual inspection reveals a generally symmetrical distribution of studies around the pooled effect size, although some slight asymmetry is noted. The formal Egger's test yielded a p-value of 0.062, which is above the conventional threshold for statistical significance ($p > 0.05$).

Figure S6 shows the funnel plot for the 'Metastatic (Stage IV)' meta-analysis (k=12 studies). Similarly, Egger's regression test yielded a non-significant p-value of 0.075.

In both analyses, the Egger's test did not provide strong statistical evidence of publication bias. However, it is notable that both p-values are borderline (close to 0.05), suggesting that some degree of small-study effect

cannot be definitively ruled out, a common limitation in meta-analyses of observational studies with high heterogeneity.

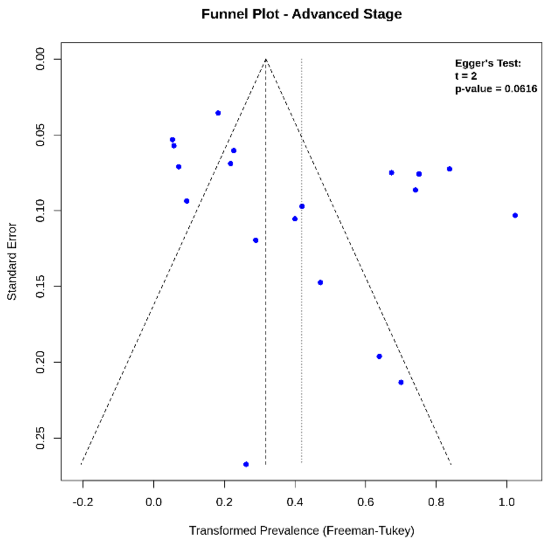

Figure S5: Funnel Plot for papers analyzed in the advanced stage meta-analysis

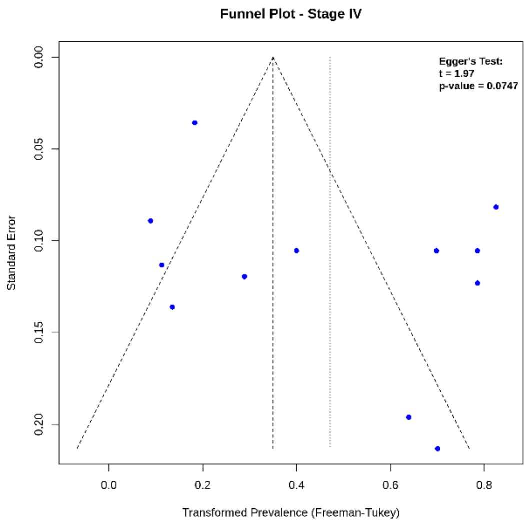

Figure S6: Funnel Plot for papers analysed in the metastatic stage meta-analysis
